# HLA-G and HLA-E specific mRNAs connote opposite prognostic significance in renal cell carcinoma

**DOI:** 10.1186/1746-1596-7-58

**Published:** 2012-05-29

**Authors:** Leos Kren, Ivo Valkovsky, Jan Dolezel, Ivo Capak, Dalibor Pacik, Alexandr Poprach, Radek Lakomy, Martina Redova, Pavel Fabian, Zdenka Krenova, Ondrej Slaby

**Affiliations:** 1 Department of Pathology, Faculty of Medicine, University Hospital Brno, Masaryk University, Jihlavska 20, Brno, 625 00, Czech Republic; 2 Faculty Hospital Ostrava, Clinic of Internal Medicine, Faculty of Medicine, University of Ostrava, Ostrava, Czech Republic; 3 Department of Oncological Urology, Masaryk Memorial Cancer Institute, Brno, Czech Republic; 4 Department of Urology, Faculty of Medicine, University Hospital Brno, Masaryk University, Brno, Czech Republic; 5 Department of Comprehensive Cancer Care, Masaryk Memorial Cancer Institute, Brno, Czech Republic; 6 CEITEC - Central European Institute of Technology, Masaryk University, Brno, Czech Republic; 7 Department of Oncological and Experimental Pathology, Masaryk Memorial Cancer Institute, Brno, Czech Republic; 8 Department of Childrens’ Oncology, Faculty of Medicine, University Hospital Brno, Masaryk University, Brno, Czech Republic

## Abstract

**Background:**

Renal cell carcinoma (RCC) is characterized by its resistance to radiotherapy and/or chemotherapy. On the other hand, it is an immunogenic tumor - it is able to stimulate antitumor responses. A prognostic significance of HLA-G expression by neoplastic cells in RCC is not well characterized; significance HLA-E expression in RCC is not characterized at all.

**Methods:**

In our study, we evaluated the expression of HLA-G and HLA-E specific mRNA transcripts produced by neoplastic cells in 38 cases of RCC and in 10 samples of normal kidney parenchyma. The results were statistically correlated with various clinico-pathological parameters.

**Results:**

We confirmed that HLA-G is downregulated in normal kidney tissue; if it is up-regulated in RCC, then it is connected to worse prognosis. On the other hand, HLA-E mRNA transcripts were present in both normal kidney tissue and RCC and their increasing concentrations counterintuitively carried better prognosis, more favorable pT stage and lower nuclear Fuhrmann’s grade.

**Conclusion:**

Considering the fact that there is known aberrant activation of HLA-G and HLA-E expression by interferons, identification of HLA-G and HLA-E status could contribute to better selection of RCC patients who could possibly benefit from more tailored neoadjuvant biological/immunological therapy. Thus, these molecules could represent useful prognostic biomarkers in RCC, and the expression of both these molecules in RCC deserves further study.

**The virtual:**

Slide(s) for this article can be found here: http://www.diagnosticpathology.diagnomx.eu/vs/7383071387016614

## Introduction

Nonclassical human leukocyte molecules G and E (HLA-G and HLA-E) were originally thought to be specifically expressed only on extravillous trophoblast - fetal tissue in contact with maternal cells which lacks MHC class I antigens. The initial described function of these molecules was the protection of fetal semiallogeneic graft from maternal allorecognition (‘pregnancy sentinels’) [1,2].

Currently, it is known that HLA-G and HLA-E exert multiple immunoregulatory functions. HLA-G is well known for its immuno-tolerogenic properties utilized by neoplastic cells in many malignancies where its expression may represent one of the various mechanisms used by tumor cells to thwart the immune response.

Immuno-modulatory molecule HLA-E function is more complex: it can act as both immuno-tolerogenic and immuno-activating molecule, depending on the type of NK cell receptor it is associated with. Furthermore, HLA-E can associate with ‘non-canonical’ peptides and utilize CD8 T-cell receptor-mediated recognition.

Renal cell carcinoma (RCC) has several subtypes, usually identifiable by H + E stain; in some subtypes, ancillary techniques are beneficial for accurate diagnosis [3]. Interestingly, some RCC express neuroendocrine markers, though their prognostic significance remains unclear [4]. RCC is characterized by its resistance to radiotherapy and/or chemotherapy; on the other hand, it is an immunogenic tumor - it is able to stimulate antitumor responses. A prognostic significance of the expression of HLA-G by neoplastic cells in RCC is not well characterized, and the significance of the expression of HLA-E in RCC is not characterized at all. Therefore, in our study, we evaluated the expression of HLA-G and HLA-E specific mRNA transcripts produced by neoplastic cells in 38 cases of renal carcinoma.

## Material and methods

### Patients

Thirty-eight patients (24 men, 14 women) diagnosed with renal cell carcinoma of a clear type at the Masaryk Memorial Cancer Institute (Brno, Czech Republic) between 2003 and 2009 were included in this study. All cases/tissues represented consecutive cases and primary diagnoses. The examination of human tissues was approved by the Hospital´s Ethical Committee. These examinations were carried out in accordance with the ethical standards laid down in an appropriate version of the 1964 Declaration of Helsinki. Patients' ages ranged from 41 to 89 years, with a median of 68 years. Histological diagnoses were established according to the guidelines of the latest WHO classification. Cases were selected according to tissue availability and were not stratified for any known preoperative or pathological prognostic factor. Clinical follow-up data in the form of annually assessed survival time was available for all patients. No patient received any neoadjuvant therapy including biological therapy/immunotherapy. The median follow-up time for all cases was 40 months and ranged from 3 to 105 months. Clinical characteristics of the patients are summarized in Table 1.

**Table 1:** Patient characteristics

**Factor**	**Number**
**Age**	
mean	68
range	41-89
**Sex**	
male	24
female	14
**Stage**	
T1 + T2	20
T3	18
**Fuhrmann grade**	
G1	6
G2	21
G3	11
**Early reccurence**	
Yes*	15
No**	23

* Recurrence-free survival = 11.5 (5-36) months.

** Follow-up = 50 (41-62) months.

Medians and 25th and 75th percentiles in parentheses.

### Tissue sample preparation and RNA purification

Under the supervision of an experienced pathologist, 48 tissue samples were collected (before start of any treatment) from surgically resected tissues - 38 samples from primary tumors and 10 from adjacent non-tumoral renal parenchyma. The inclusion criterion was at least 70% of tumor cells in the sample (confirmed by frozen section of the included tissue). All samples were immediately stored in liquid nitrogen until RNA extraction. Samples were homogenized with tissue homogenizer MM301 (Retch, Germany) in sterile conditions before total RNA isolation. Total RNA isolation procedure was performed using the RNeasy Mini Kit (Qiagen) according to manufacturer’s instructions Total RNA concentration and purity were controlled by UV spectrophotometry (A260:A280 > 2.0; A260:A230 > 1.8) using Nanodrop ND-1000 (Thermo Scientific, USA).

### Reverse transcription and real-time qPCR

cDNA was obtained by reverse transcription (Thermocycler Tgradient; Biometra). Reaction mixture containing 1ug of total RNA, random hexamers (Qiagen) and RNAse-free water was incubated at 65°C for 5 min, then cooled quickly on ice, and the following components added to 20uL final volume: 5X Reaction Buffer, Ribonuclease Inhibitor, dNTP mix, Revert Aid^TM^ MMuLV H-Reverse Transcriptase (all from Fermentas) and RNAse-free water. The mixture was incubated for 120 min at 42°C, and the reaction was stopped by heating the mixture at 95°C for 5 min and chilled on ice. The real-time PCR was performed using Applied Biosystems 7500 Sequence Detection System according to manufacturer’s recommendations. The PCR mixture, with a total volume of 20 ul, included 4 ul of the reverse transcription product, 10ul of 2X TaqMan Universal PCR Master Mix, 1 ul of 20X Primers and probe mix of the TaqMan Expression Assays for HLA-G (Hs00365950_g1) and HLA-E (Hs03045171_m1) and 5ul of RNAse-free water. Used primers amplify all of HLA-G splice variants (spanning exons 5-6). The mixture was incubated in 96-well optical plates at 50°C for 2 min, 95°C for 10 min, followed by 40 cycles of 95°C for 15 s and 60°C for 1 min. The Ct data were determined with default threshold settings. All reactions were run in duplicates, and average Ct and standard deviation values were calculated.

### Immunohistochemistry

Immunohistochemical analysis was performed by using antibodies to HLA-G (isoform G1, clone MEM-G/1, dilution 1:25, Exbio Prague, and HLA-E (clone MEM-E/02, dilution 1:25, Exbio Prague). Dilution was done by Dako REAL™ Antibody Diluent, DAKO. Formalin fixed paraffin embedded 4 μm thick whole tissue sections were cut. Tissue microarray technique was not used to avoid its possible pitfalls [5]. Following deparaffinization (pure xylen 3 × 5 min, 96% alcohol 3 × 5 min, rinsed by distilled water), inactivation of endogenous peroxidase (3%H_2_O_2_ in methanol for 10 min, rinsed by distilled water) and antigen retrieval (citrate buffer of pH 6.0 at 98°C for 20 min, cooling for 20 min, PBS buffer 3 × 5 min) followed incubations with primary antibodies. Incubations were performed in a wet chamber for 1 h at room temperature (20°C) followed by PBS buffer 3 × 5 min. Streptavidin–biotin peroxidase detection system was used based on manufacturer's instructions (EnVision + System, HRP Labeled Polymer anti Mouse, DAKO) in a wet chamber for 45 min at room temperature, washed by PBS buffer, and then visualized using 3,3′ diaminobenzidine (DAB) as a substrate (Sigma). Nuclei were counterstained by Gill's hematoxyline for 1 min followed by bluing in water for 2–3 min for optimal results. Following dehydration in a series of up-concentrated ethanol baths and clearing in xylene, the specimens were mounted in Entelan™.

### Statistical analysis

Expression data were normalized according to the expression of the reference gene GAPDH (Assay No. 402869; Applied Biosystems). Statistical differences between clinico-pathological parameters and mRNA levels were evaluated using the Mann–Whitney *U*-test between two independent groups and Wilcoxon test between paired groups and the Kruskal-Wallis test for 3 or more groups. The cut-off value for the HLA-G specific mRNAs expression was set to 75th percentile of HLA-G expression levels in RCC tissue. For the HLA-E specific mRNAs expression, the cut-off was set to 25th percentile of HLA-E expression levels in RCC tissue: owing to the relatively limited number of patients in our study, we used this strategy to select the closest quartile of ROC- identified cut-offs and use them as final cut-off values to be more illustrative for further independent studies.

The long-rank test was used to measure the differences in Kaplan–Meier curves. P-values lower than 0.05 were considered to be statistically significant. All calculations were performed with the software Statistica (StatSoft Inc., version 6.0, Tulsa, OK, USA).

## Results

**Figure 1:**
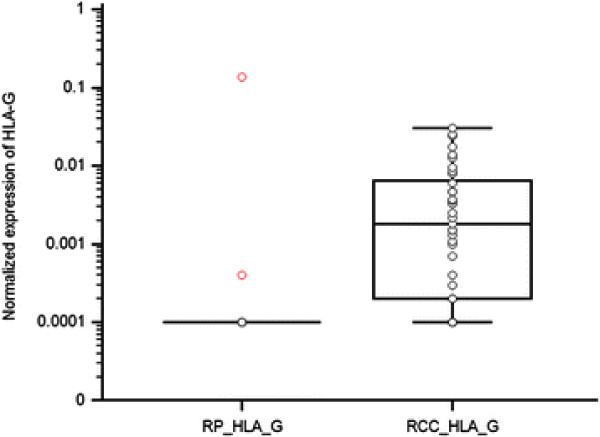
Comparison of HLA-G expression levels in RCC tissue and non-tumoral renal parenchyma (p = 0.0058, Mann–Whitney test).

**Figure 2:**
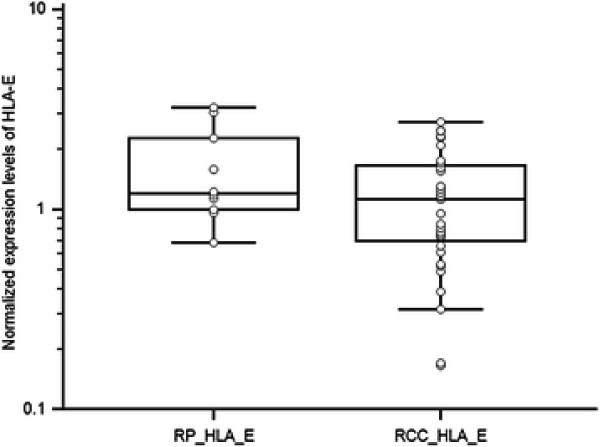
Comparison of HLA-E expression levels in RCC tissue and non-tumoral renal parenchyma (p = 0.126, Mann–Whitney test).

Expression levels of HLA-G and HLA-E in RCC tissue in comparison to non-neoplastic renal parenchyma are shown in Table 2. These results indicate that HLA-G specific mRNA transcripts were not - except of one sample - detectable in normal renal parenchyma. On the other hand, HLA-G specific mRNA transcripts were detectable in tissues of RCC in 29 cases (out of 38, i.e. 76%) and that there was a correlation between the presence of HLA-G specific mRNA transcripts in RCC tissue and normal renal parenchyma (p = 0.031 for paired samples, p = 0.006 for non-paired samples). HLA-E specific mRNA transcripts, though, were detectable in both normal renal parenchyma and tissues of RCC in all samples (Table 3), and there was no correlation between HLA-E mRNA transcripts in RCC tissue and normal renal parenchyma (p = 0.82 for paired samples, p = 0.14 for non-paired samples). Comparison of HLA-G expression levels in RCC tissue and non-tumoral renal parenchyma (p = 0.0058, Mann–Whitney test) is shown in Figure 1. Comparison of HLA-E expression levels in RCC tissue and non-tumoral renal parenchyma (p = 0.126, Mann–Whitney test) is shown in Figure 2.

**Table 2:** Expression levels of HLA-G and HLA-E in RCC tissue in comparison to renal parenchyma

	**non-paired samples**			**paired samples**		
	**RCC**	**renal parenchyma**	**p-value**	**RCC**	**renal parenchyma**	**p-value**
	**n = 38**	**n = 10**		**n = 10**	**n = 10**	
**HLA-G**	0.20*	0.01	**0.006**	0.10	0.01	**0.031**
	0.02-0.70**	0.01-0.01		0.01-0.80	0.01-0.01	
**HLA-E***x100*	1.13	1.19	**0.14**	1.30	1.19	**0.82**
	0.70-1.66	0.99-2.47		0.91-2.31	0.99-2.47	

* Medians of expression level related to GAPDH with **25th and 75th percentiles.

**Table 3:** Percentages of HLA-G and HLA-E in normal renal parenchyma and RCC tissues

	**Normal kidney (n = 10)**	**RCC (n = 38)**
HLA-G positive	1 (10%)	29 (76%)
HLA-E positive	10 (100%)	38 (100%)

**Figure 3:**
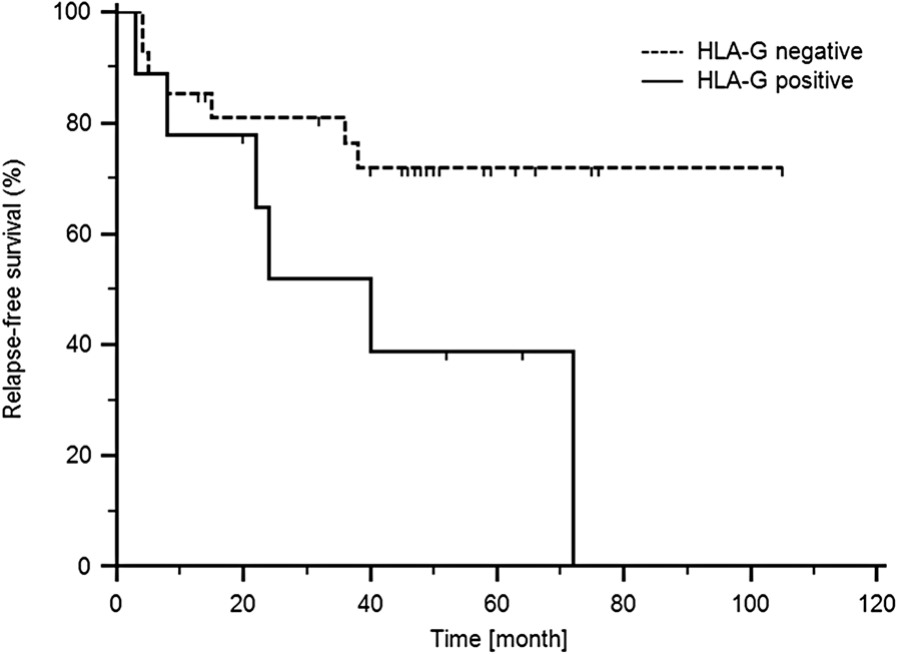
Relapse-free survival of RCC patients based on the HLA-G expression (cut off = 75th percentile of HLA-G expression levels in RCC tissue; p = 0,0401, Long-rank test).

Relapse-free survival of RCC patients based on the HLA-G expression (p = 0.0401, Log-rank test) is shown in Figure 3. These results indicate that there is a negative correlation between HLA-G expression and the length of relapse-free survival.

**Figure 4:**
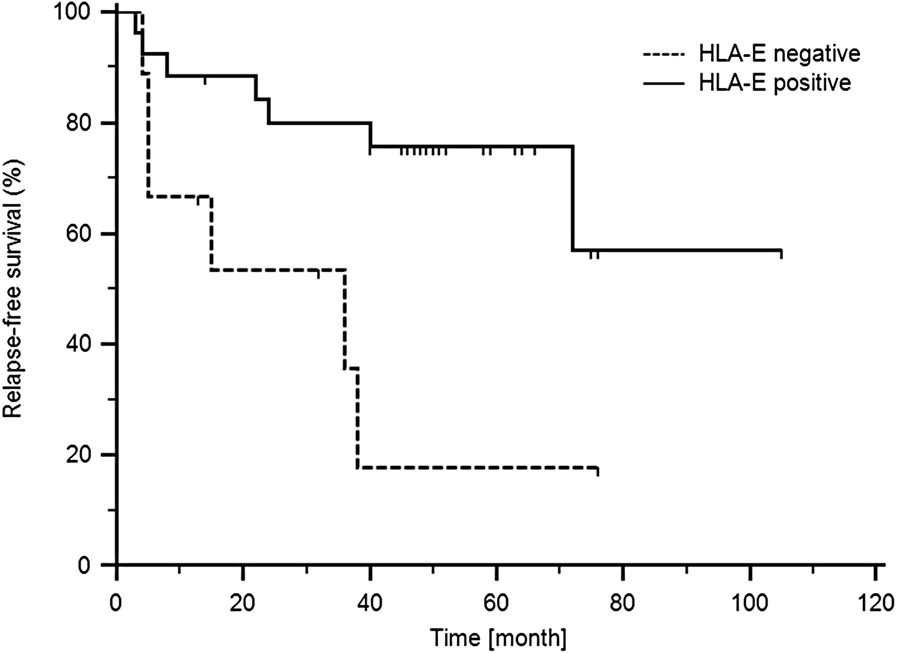
Relapse-free survival of RCC patients based on the HLA-E expression (cut off = 25th percentile of HLA-E expression levels in RCC tissue; p = 0,0115, Long-rank test).

Relapse-free survival of RCC patients based on the HLA-E expression (Log-rank test) is shown in Figure 4. These results indicate that there is a positive correlation between levels of HLA-E specific mRNA transcripts and the length of relapse-free survival.

There was no association between HLA-G expression and pT stage (p = 0.2649; Mann–Whitney test) and Fuhrmann’s grade (p = 0.1949, Mann–Whitney test).

**Figure 5:**
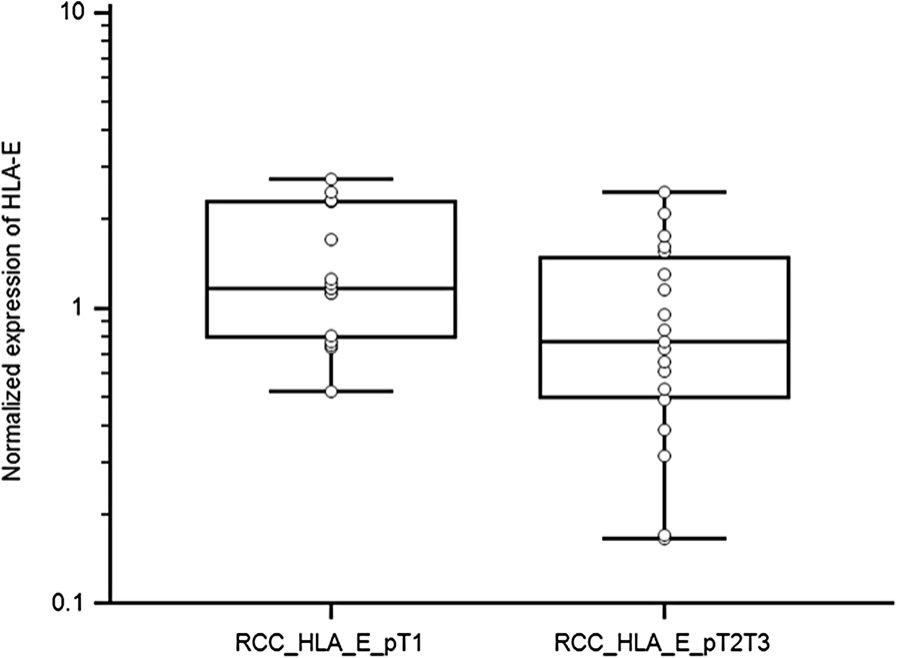
**HLA-E expression levels in RCC of different pT stage (p = 0.041, Mann–Whitney test)**.

HLA-E expression levels in RCC of different pT stage (p = 0.041, Mann–Whitney test) are shown in Figure 5 suggesting that there is a positive correlation between HLA-E expression and lower pT stage.

**Figure 6:**
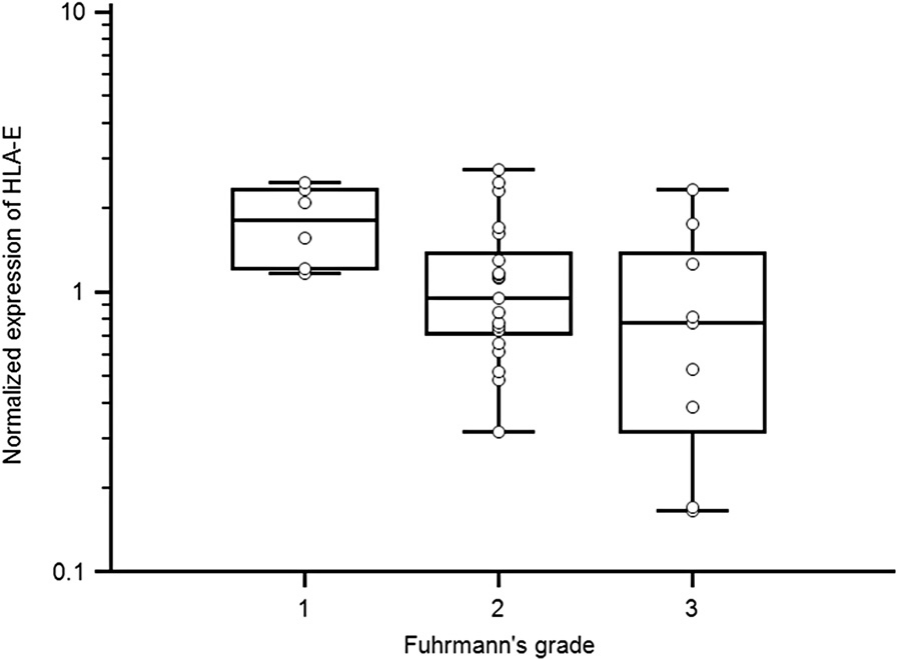
Correlation of HLA-E expression with Fuhrmann’s grade of RCC (p = 0.0423, Kruskal-Wallis test).

Correlation of HLA-E expression with Fuhrmann’s grade of RCC (p = 0.0423, Kruskal-Wallis test) is shown in Figure 6, and these findings suggest that there is a positive correlation between HLA-E expression and better Fuhrmann’s grade.

## Discussion

Originally known as ‘sentinels of pregnancy’, non-classical immuno-modulatory molecules HLA-G and HLA-E were thought to be specifically expressed only on the extravillous trophoblast. Their first described function was to counter-attack maternal immune surveillance and enable early tolerance and later acceptance of semiallogeneic fetal graft by their immune-tolerogenic properties.

The expression of these molecules by normal non-fetal tissues was later described: HLA-G proteins have been identified in thymic epithelial cells, in keratocytes from cornea and in cells of erythropoietic lineage from the bone marrow [6]. HLA-E antigen was described in B and T lymphocytes, natural killer cells, macrophages and megakaryocytes; within the normal non-lymphoid organs, expression is mainly restricted to endothelial cells [7]. However, HLA-E mRNA transcripts were identified in almost all human cells [8]. Under pathological neoplastic conditions, the HLA-G protein was identified in a variety of human malignancies.

As far as the prognostic significance of HLA-G under neoplastic conditions is concerned, a body of literature has emerged supporting the immune-tolerogenic role of HLA-G based on worse prognosis of patients with HLA-G positive tumors of different histotypes. Our results confirmed already described [9] virtual down-regulation of HLA-G expression in almost all samples of normal kidney parenchyma and its up-regulation in RCC. Although there is only one large study which specifically analyzed possible prognostic significance of HLA-G up-regulation in RCC [10] and that study *does not* indicate negative prognostic influence of HLA-G expression, our results support this role of HLA-G in RCC by negatively influencing relapse-free survival time. This observed immuno-tolerogenic effect is in accordance with both physiological functions of HLA-G and most reports that evaluate the relationship of HLA-G tumoral positivity to prognosis. Thus, aberrant HLA-G expression is found at a relatively high frequency in RCC and might participate in evasion of these tumor cells from immunosurveillance by inhibiting lysis of RCC cells by immune effector cells. Moreover, the aberrant activation of HLA-G expression in RCC cell lines due to the influence of interferons was described [9,11,12]. So, there is a tempting suggestion that considering the immunotolerogenic function assigned to date to HLA-G molecule, this induction effect of interferons could lessen or possibly even eliminate the effect of immunotherapy (and to explain, at least in part, failure to respond to immunotherapy).

Unlike HLA-G, the expression and function of HLA-E in neoplastic processes remains poorly established and understood with contradictory literature reports. Most literature data indicate worse prognosis of patients with HLA-E positive tumors of different histotypes.

[13-16] However, there are scarce reports which counterintuitively (related to physiological immuno-tolerogenic function of HLA-E in trophoblast) claim better prognosis for patients with HLA-E positive tumors [17-19]. We showed that HLA-E specific mRNA transcripts are present in both normal and malignant kidney tissue. Most importantly, our results also indicate positive influence to relapse-free survival in patients whose RCC tissues show high concentrations of HLA-E specific mRNA. Furthermore, there seems to be a positive correlation between high concentrations of HLA-E specific mRNA in neoplastic cells and better pT stage and lower Fuhrmann’s grade of RCC. The explanation of these counterintuitive effects of HLA-E in RCC could be the direct engagement of the NKG2C activation receptors instead of NKG2A inhibitory receptors [20]. Another possibility is an antigen-specific recognition through the T cells receptor expressed by NK-CTLs [18,21]. The last possibility of activation is the competitive relief of NKG2A-mediated inhibition upon HLA-E assembly with peptides from donor proteins other than HLA class I (for instance, heat peptides etc.): HLA-E molecules in complex with these signal peptides are no longer recognized by CD94/NKG2A inhibitory receptors. Such peptide interference would gradually uncouple CD94/NKG2A inhibitory recognition [22]. Thus, our results indicate that the balance between activating and inhibitory functions of multifunctional immuno-modulatory molecule HLA-E in RCC favors activation responses instead of tolerogenic effects.

An aberrant activation of HLA-E expression in various cell lines (other than RCC) due to the influence of interferons was also described [23-26]. Of note, one study [27] showed the opposing effects of various interferons (alpha vs. gamma) on effector cells: interferon-alpha stimulates expression of stimulatory NKG2D receptors and inhibits the expression of inhibitory NKG2A receptors on NK cells whereas interpheron gamma increases the expression of MICA and HLA-E cell surface proteins. We speculate that interferon gamma therapy related induction of HLA-E expression in RCC HLA-E negative patients could be one of the mechanisms of efficient interferon therapy.

**Figure 7:**
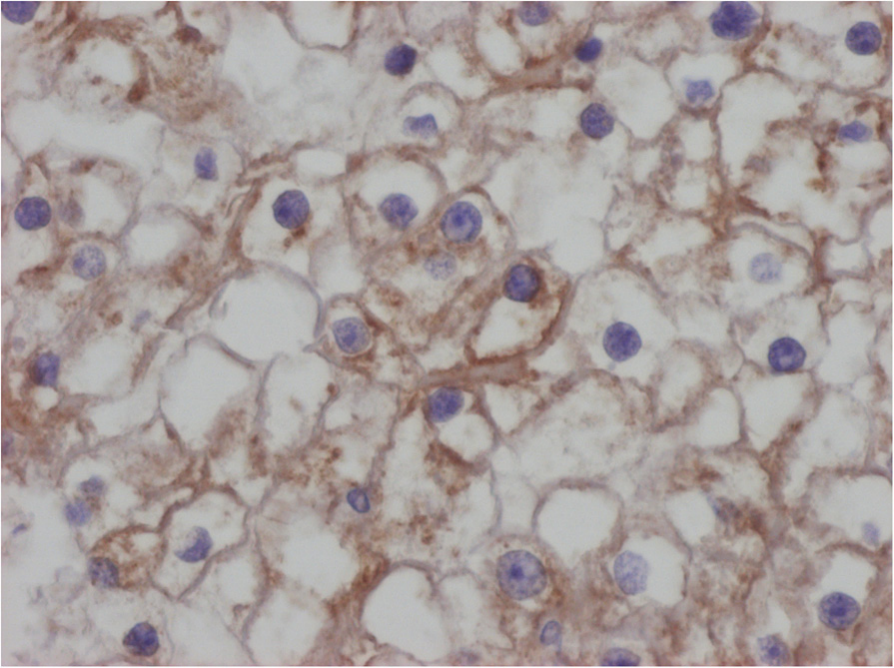
**Positivity of RCC for the HLA-G, immunohistochemistry, DAB method, clone MEM-G/1, Exbio Prague.** Analysed by Olympus BX45 microscope equipped with Olympus DP50 digital camera. Olympus Viewfinder Lite™software was used to acquire and process the image, 400×.

**Figure 8:**
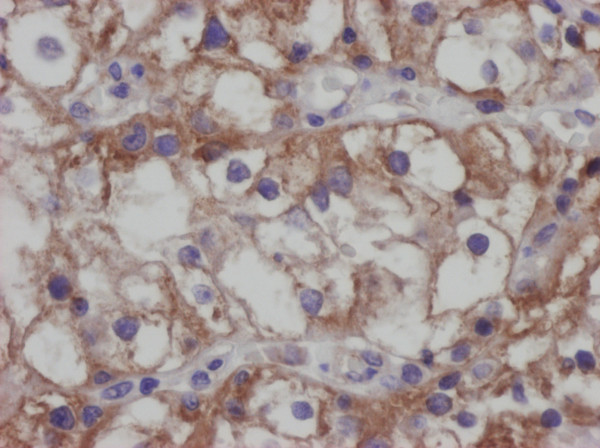
**Positivity of RCC for the HLA-E, immunohistochemistry, DAB method, clone MEM-E/02, Exbio Prague.** Analysed by Olympus BX45 microscope equipped with Olympus DP50 digital camera. Olympus Viewfinder Lite™software was used to acquire and process the image, 400×.

As far as methodology is concerned, it would be also possible to study these molecules in formalin-fixed paraffin embedded material (Figure 7 and Figure 8). That approach would have, though, three possible pitfalls: the first are possible cross-reactions of used antibodies [28,29] the second is the necessity of a subjective and possibly an inaccurate semi-quantitative evaluation of immunohistochemical slides and the third is possible regional expressional variability. Our approach eliminates all three possible pitfalls.

To summarize, for the first time, we described negative prognostic significance of HLA-G transcripts but positive prognostic significance of HLA-E transcripts in RCC. Thus, these molecules could represent useful prognostic biomarkers in RCC. Further, we described for the first time positive influence of HLA-E transcripts to better pT stage and to higher nuclear Fuhrmann’s grade. In our study, we also confirmed the absence of HLA-G specific mRNA and the presence of HLA-E specific mRNA in normal renal parenchyma. Further work is required to investigate these mechanisms in the context of activation-inhibition responses of tumor immunosurveillance. Characterization of HLA-G and HLA-E status could thus contribute to better prediction and monitoring of RCC.

## Conclusions

The role of immuno-modulatory nonclassical molecules HLA-G and HLA-E in the development and in the clinical course of RCC is still not well characterized.

Here, we described that HLA-G is downregulated in normal kidney tissue; if it is up-regulated in RCC, then it carries the worse prognosis. On the other hand, HLA-E mRNA transcripts were present in both normal kidney tissue and RCC and their increasing concentrations counterintuitively carried better prognosis, more favorable pT stage and lower nuclear Fuhrmann’s grade. These molecules could represent useful prognostic biomarkers in RCC. Moreover, considering the fact that there is known aberrant activation of HLA-G and HLA-E expression by interferons, then identification of HLA-G and HLA-E status could have an impact on the design of T and NK cell-based immunotherapies in this disease and contribute to better selection of RCC patients who could possibly benefit from more tailored neoadjuvant biological/immunological therapy.

The expression of both these molecules and their roles in RCC deserve further study.

## Abbreviations

RCC, Renal cell carcinoma; HLA-G, Human Leukocyte Antigen G; HLA-E, Human Leukocyte Antigen E; H + E, Hematoxylin and eosin stain; PCR, Polymerase chain reaction.

## Competing interests

The authors declare no competing interests

## Authors’ contributions

IV organized data for the manuscript, JD, IC, DP collected tissues, AP, RL, MR and ZK participated in data organization and manuscript drafting, PF performed histopathological diagnoses, OS performed molecular and statistical analyses and LK provided project design, coordinated the study and writing of the manuscript. All authors read and approved the final manuscript.
